# SARS-CoV-2 Vaccine Hesitancy in a Sample of US Adults: Role of Perceived Satisfaction With Health, Access to Healthcare, and Attention to COVID-19 News

**DOI:** 10.3389/fpubh.2021.665724

**Published:** 2021-04-29

**Authors:** Sarah Bauerle Bass, Maureen Wilson-Genderson, Dina T. Garcia, Aderonke A. Akinkugbe, Maghboeba Mosavel

**Affiliations:** ^1^Department of Social and Behavioral Sciences, Temple University College of Public Health, Philadelphia, PA, United States; ^2^Siminoff Research Group, Temple University College of Public Health, Philadelphia, PA, United States; ^3^Department of Health Behavior and Policy, Virginia Commonwealth University, Richmond, VA, United States; ^4^Department of Dental Public Health and Policy, Virginia Commonwealth University, Richmond, VA, United States

**Keywords:** COVID-19, SARS-CoV-2, vaccine hesitancy, satisfaction with healthcare access, satisfaction with health

## Abstract

Understanding which communities are most likely to be vaccine hesitant is necessary to increase vaccination rates to control the spread of SARS-CoV-2. This cross-sectional survey of adults (*n* = 501) from three cities in the United States (Miami, FL, New York City, NY, San Francisco, CA) assessed the role of satisfaction with health and healthcare access and consumption of COVID-19 news, previously un-studied variables related to vaccine hesitancy. Multilevel logistic regression tested the relationship between vaccine hesitancy and study variables. Thirteen percent indicated they would not get vaccinated. Black race (OR 2.6; 95% CI: 1.38–5.3), income (OR = 0.64; 95% CI: 0.50–0.83), inattention to COVID-19 news (OR = 1.6; 95% CI: 1.1–2.5), satisfaction with health (OR 0.72; 95% CI: 0.52–0.99), and healthcare access (OR = 1.7; 95% CI: 1.2–2.7) were associated with vaccine hesitancy. Public health officials should consider these variables when designing public health communication about the vaccine to ensure better uptake.

## Introduction

Vaccine hesitancy is a public health threat and its underlying causes could significantly affect successful uptake of a SARS-CoV-2 vaccine. Along with current preventive measures, a vaccine could alter the pandemic's longevity, but given estimates that a potential vaccine would need to be at least 80% effective and reach at least 75% of the population (1), understanding who is most likely to be hesitant is critical to understand how best to intervene and encourage vaccination. Vaccine acceptance is a complex decision, swayed by personal health beliefs, interpersonal influences, and mistrust of the healthcare system and those developing and distributing vaccines. Most current vaccine hesitancy research focuses on parents; a recent study indicates about 6.1% of parents are hesitant and of those, 67.5% report deferring or refusing vaccinations for their children (2). Less is known, however, about adult vaccine hesitancy. The few SARS-CoV-2 vaccine hesitancy studies look at sociodemographic characteristics of hesitators or vaccine hesitancy more generally (3, 4), not at personal beliefs about health and healthcare access or consumption of COVID-19-related news. The purpose of this study was to assess characteristics of adults who say they would not get a COVID-19 vaccine in those living in three U.S. cities and how personal perceptions of health and healthcare access, as well as reported consumption of news, may contribute to vaccine hesitancy.

## Methods

### Study Sample

Cross-sectional online surveys were collected between May 15 and July 6, 2020 from a non-probability sample of adults recruited from New York City, NY, Miami, FL, and San Francisco, CA through the Qualtrics platform. Qualtrics has large market research panels that consist of people who have agreed to participate in surveys; they are then selected based on survey criteria. Cities were chosen for high proportions of COVID-19 cases and the existence of differing shut-down restrictions. Eligibility criteria included being a minimum age of 18, attendance at religious services at least twice a month, and residency in one of the three cities. Primary analysis assessed use of alternative religious worship services, social support and quality of life outcomes in relation to COVID-19, but other variables allowed for secondary analysis on vaccine hesitancy. All participants of the survey were included in analysis. A total of 501 people participated and provided informed consent. Human subjects' approval was received from Virginia Commonwealth University (HM20019222).

### Measures

A survey developed by the researchers consisted of sociodemographic variables that included age, education, gender, self-reported race and ethnicity, income. Independent variables included: 1. *Inattention to COVID-19 news* (“How closely have you been following the news…about the COVID-19 outbreak?”; 4-point scale; 0 = Not closely at all, 3 = Very closely; reverse coded); 2. *Satisfaction with health* (“How satisfied were you with your health before the stay at home recommendations?”; 5-point scale; 1 = Very dissatisfied, 5 = Very satisfied); 3. *Satisfaction with access to healthcare* (“How satisfied were you with your access to health services before the stay at home recommendations?”; 5-point scale; 1 = Very dissatisfied, 5 = Very satisfied; reverse coded). The outcome variable was *vaccine hesitancy* (“If a vaccine…were available, how likely would you be to be vaccinated?”; 0 = Very likely/somewhat likely, 1 = not at all/not too likely).

### Statistical Analysis

Descriptive statistics were computed for sociodemographic variables comparing participants who would avoid a vaccine to those who would not (chi-square and *t*-test). Multilevel logistic regression was used to assess the relationship between vaccine hesitancy and all other variables while controlling for nesting of participants within city. Associations are presented as odds ratios (OR) with 95% CI. Data analysis was conducted in SAS v. 9.4 (SAS Institute, Cary, NC). *Post-hoc*-power analysis was also completed to assess ability to detect differences using either *t*-tests or Fisher exact test of proportions.

## Results

The sample was 64% male, 76% White, and 58% were between the ages of 30 and 64. Thirteen percent of the sample indicated they would not be willing to be vaccinated (Table 1). Vaccine avoiders were more likely Black or African American (χ = 30.5, *p* < 0.001), younger (χ = 28.1, *p* < 0.001), female (χ = 7.4, *p* < 0.01), have lower education (χ = 20.5, *p* < 0.001) and income levels (χ = 43.1, *p* < 0.001), and were not from San Francisco (χ = 7.3, *p* < 0.05). They were also more likely to ignore COVID-19 news (*t* = 3.77; *p* < 0.0003) and be less satisfied with their health (*t* = 3.54; *p* < 0.0007) and their access to health care (*t* = 4.72, *p* < 0.0001). Other variables, such as political affiliation or religion, were not found to be significant and therefore not included in the regression analysis.

**Table 1:** Characteristics of sample by willingness to have SARS-CoV-2 vaccine.

	**Total *N* = 501**	**Accept vaccine *N* = 436 (87%)**	**Avoid vaccine *N* = 65 (13%)**	***X*^**2**^ (*df*)**	***p*-value**
	***N* (%)**	***N* (%)**	***N* (%)**		
**Age**					
18–29	116 (23.1)	87 (75.0)	29 (25.0)	28.13 (3)	***p*** **<** **0.001**
30–49	198 (39.5)	188 (95.0)	10 (5.0)		
50–64	91 (18.2)	75 (82.4)	16 (17.6)		
65+	96 (19.2)	86 (89.6)	10 (10.4)		
**Gender**					
Male	318 (63.7)	287 (90.3)	31 (9.7)	7.42 (1)	***p*** **<** **0.01**
Female	181 (36.3)	148 (81.8)	33 (18.2)		
**Education**					
< High School	6 (1.2)	5 (83.3)	1 (16.7)	20.5 (3)	***p*** **<** **0.001**
High School	31 (6.2)	21 (67.7)	10 (32.3)		
Some College	115 (22.9)	92 (80.0)	23 (20.0)		
College graduate of higher	349 (69.7)	318 (91.1)	31 (8.9)		
**Income**					
<10–29,999 k	69 (13.8)	47 (68.1)	22 (31.9)	43.1 (4)	***p*** **<** **0.001**
30–59,999	82 (16.4)	63 (76.8)	19 (23.2)		
60–99,999	127 (25.3)	115 (90.6)	12 (9.4)		
100–149,999	105 (21.0)	97 (92.4)	8 (7.6)		
>150,000	118 (23.5)	114 (96.6)	4 (3.4)		
**Latino**					
No	414 (82.6)	365 (88.2)	49 (11.8)	2.74 (1)	*p* < 0.10
Yes	87 (17.4)	71 (81.6)	16 (18.4)		
**Race**					
Black	59 (11.8)	38 (64.4)	21 (35.6)	30.5 (3)	***p*** **<** **0.001**
White	382 (76.2)	344 (90.1)	38 (9.9)		
Asian	34 (6.8)	30 (88.2)	4 (11.8)		
Other/Multi	26 (5.2)	24 (92.3)	2 (7.7)		
**City**					
NYC	225 (44.9)	200 (88.9)	25 (11.1)	7.34 (2)	***p*** **<** **0.05**
Miami	175 (34.9)	143 (81.7)	32 (18.3)		
San Francisco	101 (20.2)	93 (92.1)	8 (7.9)		
	**M (*****sd*****)**	**M (*****sd*****)**	**M (*****sd*****)**	***t*****-test**	***p*****-value**
Not following COVID news	0.39 (0.61)	0.34 (0.54)	0.75 (0.86)	3.77	**0.0003**
Satisfaction with health status	4.04 (0.88)	4.1 (0.81)	3.46 (1.1)	3.54	**0.0007**
Dissatisfaction with access to healthcare	0.25 (0.56)	0.20 (0.49)	0.60 (0.80)	4.72	**0.0001**

*Bold items are statistically significant*.

Results of the logistic regression affirm that higher income (OR = 0.64; 95% CL: 0.50–0.83) and greater satisfaction with one's health (OR 0.72; 95% CL: 0.52–0.99) are negatively associated with vaccine avoidance; Black or African American race (OR 2.6; 95% CL: 1.38–5.3), dissatisfaction with access to health care (OR = 1.7; 95% CL: 1.1–2.7) and inattention to news coverage of COVID-19 (OR = 1.6; 95% CL: 1.1–2.5) are positively associated with vaccination avoidance (Table 2).

**Table 2:** Logistic regression—associations with vaccine avoidance.

	**B (se)**	**OR (95% CL)**	***p*-value**
Intercept	0.41 (0.97)		
Age	0.02 (0.14)	1.02 (0.77–1.35)	0.910
Gender	0.04 (0.32)	1.04 (0.56–1.93)	0.902
Education	−0.11 (0.15)	0.90 (0.66–1.21)	0.480
Income	−0.44 (0.13)	0.64 (0.50–0.83)	**0.001**
Race (Black)	0.96 (0.36)	2.60 (1.28–5.29)	**0.008**
Satisfaction with health status	−0.33 (0.16)	0.72 (0.52–0.99)	**0.043**
Unsatisfied with Health Care Access	0.55 (0.23)	1.73 (1.2–2.72)	**0.018**
Inattention to COVID news	0.48 (0.22)	1.62 (1.05–2.5)	**0.030**

*Bold items are statistically significant*.

*Post-hoc* power analysis indicated the sample was sufficient to detect difference in means by study variables, with power ranging from 0.952 to 0.99.

## Discussion

Results of this study go beyond demographic characteristics of vaccine hesitancy and include perceived satisfaction with health and health care access, as well as attention to COVID-19-related news. This is significant as those who are dissatisfied with their health or healthcare access may also have less trust for the institutions providing a vaccine. This may stem from previous experiences of discrimination of health services or expectations of care, termed “a pandemic on a pandemic” by Laurencin and Walker in the context of COVID-19 (5), where mistrust of healthcare may stem from larger societal issues, such as unfair policing and systemic racism. Indeed, national demonstrations protesting George Floyd's murder occurred during data collection, likely increasing feelings of mistrust. However, mistrust of healthcare institutions is not specific to only racial or ethnic minoritized groups and may be important to address in broader populations to increase acceptance of a SARS-CoV-2 vaccine (6). In this case, dissatisfaction of healthcare access may serve as a proxy for mistrust but may also be a larger concept that goes beyond the more common understanding of the association between trust and vaccine hesitancy.

Similarly, non-attention to COVID-19-related news was found to be independent of political beliefs (which was not seen to be significant) or race, making it an important variable to consider when thinking about how best to reach vaccine hesitant groups. A study by Calvillo et al. showed that political conservatism was associated with perceiving less personal vulnerability to the virus and believing the severity of the virus was low. These respondents also believed that the media had exaggerated the impact of COVID-19, impacting their overall knowledge of COVID-19 and how it is spread (7). This inattention to information may spill over to beliefs about vaccination, making this sub-group a potential important target for vaccination related information.

More respondents in this sample were “very likely” or “somewhat likely” to say they would get vaccinated compared to other recent surveys (3). This may in part be due to the sample's makeup. A previous study on quarantine intentions during a hypothetical avian flu outbreak found that that those who reported higher religiosity were also more willing to comply with public health directives (8). However, there is some evidence that those from certain religious backgrounds are less likely to accept some vaccines (9). However, we did not find religious differences by vaccination status and this sample was also from severely affected areas and a majority indicated they knew someone with COVID-19. This personal connection may have influenced the risk perception of respondents since being more aware of the risk and fear of personal impact have both been identified as key factors in elevating perceived risk (10).

Overall, these results provide insights on SARS-CoV-2 vaccine hesitancy beyond demographics. Many of the groups most likely to be at higher risk of the negative consequences of COVID-19 are also less likely to say they will get vaccinated (11). Importantly, we may also need to look to those who are dissatisfied with their health or healthcare access or are not consuming news about COVID-19 as being more vaccine hesitant. While these characteristics may overlap with demographics, understanding these unique perceptions may broaden our efforts in reaching those with vaccination information.

Limitations of the study include a narrower cross-sectional sample than the general public in that the survey respondents consisted only of those who indicated they attended religious services. Thus, results may not be generalizable to a broader, non-religious population. There are also inherent biases in an online sample; Qualtrics uses participants who are part of existing user panels, which may not be representative. However, we found good distribution by demographics and geographic region, indicating robustness in analysis. Finally, the study data provide a limited yet important snapshot in time in the epidemic, prior to vaccinations being a reality. Now that vaccines are available, further research could elucidate if these factors are still seen as important correlates to vaccine hesitancy.

## Conclusions

With the recent approval of three SARS-CoV-2 vaccines and their distribution to front-line workers and the general public in the United States (12), it will be imperative for public health and healthcare entities to prioritize vaccination to those most vulnerable to infection. It will be important not to assume that individuals in these groups will readily acquiesce to vaccination as they also appear to be most likely to be vaccine hesitant. Following guidelines such as the WHO “Guide to Tailoring Immunization Programmes” (13) framework may be one way to think about targeting COVID-19 vaccination communication. Immediate planning for how best to communicate about the benefits and address concerns about perceived risks of vaccination for these at-risk groups will be a way to ensure that negative health effects of COVID-19 are mitigated.

## Data Availability Statement

The raw data supporting the conclusions of this article will be made available by the authors, without undue reservation.

## Ethics Statement

The studies involving human participants were reviewed and approved by Virginia Commonwealth University. The patients/participants provided their written informed consent to participate in this study.

## Author Contributions

SB wrote the brief with significant contribution by MM. MW-G analyzed the data, wrote the methods, and results sections. AA and DG contributed to the design of the survey measure and study design and provided input on the brief. All authors contributed to the article and approved the submitted version.

## Conflict of Interest

The authors declare that the research was conducted in the absence of any commercial or financial relationships that could be construed as a potential conflict of interest.
